# Does parenting help to explain socioeconomic inequalities in children's body mass index trajectories? Longitudinal analysis using the Growing Up in Scotland study

**DOI:** 10.1136/jech-2015-206616

**Published:** 2016-04-07

**Authors:** Alison Parkes, Helen Sweeting, Robert Young, Daniel Wight

**Affiliations:** MRC/CSO Social and Public Health Sciences Unit, University of Glasgow, Glasgow, UK

**Keywords:** OBESITY, INEQUALITIES, CHILD HEALTH

## Abstract

**Background:**

Pathways leading to socioeconomic inequalities in young children's body mass index (BMI) are not well understood. This study examined whether parenting related to the physical and social context of children's food consumption helped to explain associations between maternal educational level and child BMI trajectory.

**Methods:**

The study used data from 2957 families in a nationally representative birth cohort study surveyed from 2004 to 2011, with child BMI z-score measured 3 times (46, 70 and 94 months). Latent growth curve models examined associations between maternal education and BMI z-score trajectory, exploring mediating effects of parenting (positive mealtime interaction, informal meal setting and child bedroom TV) and unhealthy child diet.

**Results:**

After adjusting for maternal BMI, maternal education predicted increased inequality in child BMI z-score trajectory slope over the study period. The slope index of inequality coefficient for maternal education, that is, the change in z-score associated with the lowest relative to the highest maternal education level, was 0.17, p<0.001. Indirect effects of lower maternal education on steeper BMI trajectory via parenting and/or unhealthy diet represented 89% of the total effect. Pathways via parenting and then unhealthy diet accounted for 68% of significant indirect pathways, with the remainder via unhealthy diet only. Bedroom TV was the most important parenting pathway, followed by informal meal setting.

**Conclusions:**

Pathways via parenting helped to explain the emergence of inequalities in young children's BMI related to maternal education. Interventions targeting parental provision of child bedroom TV and informal meal setting might reduce these inequalities.

## Background

Parental socioeconomic position (SEP) is inversely associated with childhood obesity in high-income countries. A review found that parental education is the SEP indicator most strongly associated with childhood obesity, even after controlling for maternal body mass index (BMI).^1^ Inequalities in children's BMI according to maternal education appear after about 4 years of age and widen thereafter,^2^ ^3^ but pathways underlying the development of inequalities are not currently well understood. It is well known that children of lower SEP, like their parents, have higher fat and sugar intake.^4^ However, we have only fragmented knowledge of how socioeconomic variation in family life shapes children's diet, and how this may translate into socioeconomic variation in children's BMI.^5^ ^6^ This study addresses our gap in knowledge surrounding key influences, focusing on aspects of family life with young children likely to reflect important elements of parental choice and control, and which may be potentially modifiable. There has been much interest in parental feeding practices directly concerned with children's eating.^7^ Some practices vary according to parental SEP, but many do not appear strongly linked to children's BMI.^6^ Here, we are concerned with more general aspects of parental provision and behaviour, referred to collectively as ‘parenting’. We select aspects of parenting related to the physical and social context of children's food consumption that have been identified elsewhere as being patterned according to parental SEP and associated with children's unhealthy diet and/or BMI. Our study aims to develop a more complete model for the emergence of SEP-related inequalities, constructing plausible pathways from parental SEP to child BMI trajectory via parenting and its effects on unhealthy diet, and assessing their likely importance.

Family mealtimes have been a primary focus of research interest in relation to children's diet and weight gain. Eating together is a less-established practice among lower SEP families,^8^ and might help explain inequalities in child weight gain. Nonetheless, while shared family meals have been linked to a healthy diet,^9^ reviews find associations between family meal frequency and childhood overweight are weak and inconsistent.^9^ ^10^ Given these findings, and that simply knowing whether families eat together tells us little about family processes protecting against weight gain,^11^ it seems important to consider a broader range of contextual aspects of children's food consumption at home. One context already receiving considerable research attention is a TV-orientated home environment. Two aspects of this, eating meals while watching TV and the child having a TV in her/his bedroom, are more common among low SEP groups.^12^ ^13^ A large multinational European study of 2–9-year-olds found both factors were independently associated with a diet high in fats and sugars, and with overweight, over and above time spent watching TV.^14^ This suggests the possibility of two different dietary pathways to overweight arising from a TV-orientated context of food consumption, involving family mealtimes in front of a TV and the child snacking between meals while watching a bedroom TV. However, the extent to which TV's effects on children's diet translates into SEP-related BMI inequalities has not yet been established.

Other contextual aspects of family food consumption have been less extensively researched. There are indications that an informal mealtime setting—not being seated at a table—has associations with children's unhealthy diet^15^ and BMI^16^ that are independent of TV use at meals. In addition, the quality of social interaction at mealtimes is associated with parental regulation of children's eating habits.^17^ It has been linked to child BMI in one cross-sectional study,^11^ although another study taking greater account of mealtime setting found associations only with adult BMI.^16^ Lower socioeconomic groups are less likely to eat at a table,^18^ with fewer positive interactions at mealtimes (such as sharing feelings, discussing family events, joking) compared with high SEP groups.^11^ Again, it is not known how social variation in these parenting behaviours translates into SEP-related inequalities in children's BMI.

This study uses a nationally representative cohort sample to examine associations between maternal education and children's BMI trajectory over a 4-year period, from 46 to 94 months: a critical developmental period when SEP inequalities are likely to emerge and increase.^2^ ^3^ It explores whether variation in BMI trajectory according to mothers' education is mediated via the effect of parenting on children's unhealthy diet. We investigate the following hypotheses:

- Lower maternal education will be associated with three aspects of parenting: less positive mealtime interaction, an informal mealtime setting (watching TV, and/or sitting away from a dining area) and bedroom TV;
- All aspects of parenting will have positive associations with unhealthy child diet;
- Unhealthy child diet will be associated with a steeper BMI trajectory, as evidenced in recent longitudinal research.^19^

## Methods

### Data set

Data were from the first birth cohort of the Growing Up in Scotland study, a nationally representative cohort of families with children born between June 2004 and May 2005. Details of the sampling framework are provided elsewhere.^20^ Families were first interviewed (N=5217) when children were 10 months old, and followed up annually until 70 months and then again at 94 months. Data collection was subject to medical ethical review by the Scotland ‘A’ MREC committee.

### Outcome measure: child BMI z-score

Height and weight measurements were obtained by trained researchers when the child was 46, 70 and 94 months old. BMI (weight (kg)/height (m)^2^) was calculated, and standardised BMI z-scores were derived from 1990 British growth reference charts.^21^ Measures three SDs or more from the mean were treated as potentially unreliable, and recoded as missing (N=20 at 46 months, and N=24 at 70 and 94 months).

### Main predictor: maternal educational level

This was based on maternal reports and classified into five groups, using the Scottish Credit and Qualifications Framework.^i^ These were: (1) degree-level qualifications; (2) advanced vocational qualifications (higher national certificate or equivalent); (3) upper level secondary school qualifications, leading to university entry (higher or equivalent); (4) lower level secondary school qualifications (standard grades or equivalent); (5) no qualifications. A slope index of inequality^22^ (SSI) in maternal education was calculated, with zero representing the *highest* ranking SEP and one the *lowest* possible SEP rank.

### Potential mediators

A latent variable approach was used to model mediators, offering advantages in terms of accurate estimation of indirect effects and allowance for non-normality.^23^ *Unhealthy diet* was measured at 58 months using six indicators: (1) skipping breakfast on a typical day; number of different types of (2) vegetable and (3) fruit consumed the previous day; and how often the child consumed (4) sweets and chocolates; (5) crisps; and (6) soft drinks, excluding diet or sugar-free drinks. Response options were: skipping breakfast, no (0), yes (1); vegetable and fruit consumption open-ended response (range 0–8); consumption of sweets, crisps and soft drinks, eight-point scale from (1) never to (8) more than once a day. Two indicators of *positive mealtime interaction* represented mothers' views at 58 months on whether ‘mealtimes are enjoyable for everyone’ and ‘mealtimes give us time to talk to each other’; with responses from (1) never to (4) mostly. Indicators of *informal mealtime setting* included eating a main meal watching TV (58 months); and the room where the child typically ate a main meal (22 and 58 months). Eating while watching TV was coded from (1) never to (4) often. For room information, seven response options were grouped into (0) rooms with a dining or food preparation area (kitchen, dining room, combined living/dining room) or (1) those without a dining area (living room, bedroom, other room). Child *bedroom TV* was measured using two indicators at 46 and 58 months, coded as (0) ‘no’ or (1) ‘yes’.

### Covariates

These included child gender, exact age in months at first time point of data collection (mean age 46 months), maternal ethnic group (White or Minority) and maternal BMI at child aged 70 months.

### Analysis

In total, 3196 families were interviewed at all relevant time points from 10 to 94 months. The analysis was restricted to 2957 cases where the child was a singleton birth, the natural mother was interviewed on all occasions and child BMI information was available at one or more measurement points.

Initial visualisation of the data suggested a linear decline in BMI z-scores over the study period. A linear latent growth curve model of mean child BMI was constructed using Mplus V.7.3,^24^ and the association between maternal education and BMI trajectory was explored, before and after adjusting for maternal BMI. Missing data were handled using Full Information Maximum Likelihood.

A path model was constructed to examine mediation from maternal education to child BMI trajectory via parenting and then child unhealthy diet, as well as via unhealthy diet only. We also included alternative pathways via parenting directly to child BMI (ie, not via unhealthy diet), as well as allowing for a direct effect of maternal education on child BMI (ie, not via any mediator). Mediation was assessed using the Mplus Model Indirect function, which decomposes the total effect of maternal education into direct and indirect effects. Bias-corrected bootstrapped estimates were produced following recommended practice.^25^ As software constraints did not permit the complex survey design and weights in this model, a sensitivity analysis used complex survey features and saved factor scores of the latent mediator variables.

## Results

Information on child obesity levels and risk factors is provided in table 1. With the exception of child gender and maternal ethnic group, significant differences were found for all measures according to the level of maternal education.

**Table 1:** Child obesity and risk factors for the total sample, and by maternal educational level

		Maternal education level				
	Total sample	Degree level	Advanced vocational	Upper level secondary school	Lower level secondary school	No qualifications
*N unweighted (weighted)*	*2957*	*972 (770)*	*402 (367)*	*658 (645)*	*706 (832)*	*152 (242)*
Child obesity (46 months)	11.1	8.5	7.9	14.3	10.5	17.2
Child obesity (70 months)	9.9	6.1	8.7	11.9	11.5	13.2
Child obesity (94 months)	13.1	8.2	11.2	15.5	14.8	19.1
Child female gender	48.4	47.7	50.5	47.8	48.8	45.8
Maternal minority ethnic group	3.0	3.4	3.4	2.8	1.8	4.1
Maternal obesity	16.1	13.1	14.8	16.9	16.6	24.0
Family income—lowest quintile	24.7	4.4	17.6	21.0	39.8	59.1
Area deprivation—highest quintile	23.4	8.1	21.2	21.8	31.4	52.7
Unhealthy diet						
Sweets/chocolate (58 months)	5.99 (0.04)	5.60 (0.05)	5.91 (0.08)	5.95 (0.06)	6.26 (0.06)	6.56 (0.13)
Crisps (58 months)	4.97 (0.04)	4.39 (0.05)	4.95 (0.08)	4.96 (0.06)	5.29 (0.07)	5.76 (0.16)
Soft drinks (58 months)	4.45 (0.10)	3.74 (0.12)	4.50 (0.17)	4.56 (0.15)	4.84 (0.16)	5.07 (0.28)
Vegetables (58 months)	1.60 (0.03)	1.98 (0.05)	1.71 (0.07)	1.59 (0.05)	1.33 (0.05)	1.15 (0.11)
Fruit (58 months)	1.96 (0.03)	2.23 (0.04)	2.10 (0.06)	2.03 (0.05)	1.69 (0.05)	1.58 (0.09)
Skip breakfast (58 months)	4.8	1.1	2.7	3.1	5.3	22.7
Informal meal setting						
Main meal in non-dining area (22 months)	36.2	17.0	30.2	38.0	48.8	65.4
Main meal in non-dining area (58 months)	32.2	13.5	27.1	33.3	45.2	64.4
Eat main meal watching TV (58 months)	2.20 (0.03)	1.83 (0.04)	2.16 (0.06)	2.21 (0.05)	2.38 (0.05)	2.83 (0.10)
Positive mealtime social interaction						
Mealtimes are enjoyable (58 months)	3.27 (0.02)	3.34 (0.03)	3.34 (0.05)	3.28 (0.04)	3.23 (0.03)	3.07 (0.09)
Mealtimes give us time to talk (58 months)	3.37 (0.02)	3.49 (0.03)	3.44 (0.04)	3.46 (0.03)	3.27 (0.04)	3.03 (0.09)
Bedroom TV						
Bedroom TV (46 months)	45.8	14.8	43.0	50.2	63.1	81.1
Bedroom TV (58 months)	51.6	18.8	52.2	56.5	69.5	83.5

With the exception of italicised, figures show percentages or means with SEs in parentheses, and are adjusted for survey design and weights. Sample sizes for maternal education groups do not sum to 2957 due to missing information for maternal education. All differences by maternal education level were significant at p<0.001, with the exception of child gender and maternal ethnicity.

**Figure 1:**
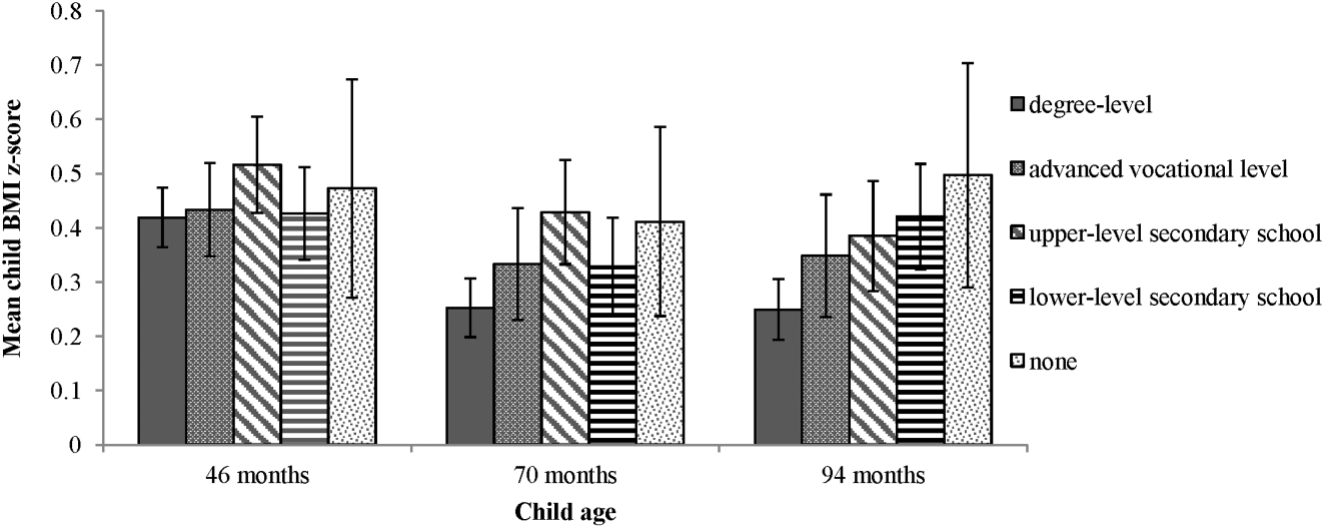
Child BMI at ages 46, 70 and 94 months according to maternal educational level. Note: bars show 95% CIs. BMI, body mass index.

Obesity levels in the sample (≥95th BMI centile, according to British growth charts) increased slightly from 11% at 46 months to 13% at 94 months,^ii^ although mean BMI z-score fell from 0.45 (95% CI 0.40 to 0.49) to 0.36 (0.31, 0.41). The z-score decrease is consistent with declining raw BMI scores across this age range in other UK studies,^26^ ^27^ together with flat corresponding sections of 1990 BMI growth curves.^28^ Figure 1 shows mean child BMI z-score at each time point according to level of maternal education, and indicates increasing inequalities in mean BMI z-score over the 4-year period. At 46 months, there were few clear differences between education groups (only one contrast, children of mothers with degree-level qualifications vs those with upper level school qualifications, was statistically significant to p<0.05). By 94 months, children whose mothers had degree-level education had significantly lower mean BMI z-scores than all other groups except mothers with advanced vocational qualifications. BMI z-scores regressed on maternal education ranked scores (SSI) confirm this picture of increasing inequalities (coefficients 0.10, p=0.210; 0.15, p=0.040 and 0.27, p=0.001 at 46, 70 and 94 months, respectively).

A latent growth model of BMI z-score had a mean intercept of 0.45 (at 46 months, p<0.001) and a negative slope of **−**0.06 (representing the change over the entire 46–94-month period, p<0.001). The SSI for lower maternal education was not associated with child BMI intercept at 46 months (table 2), but it had a positive association with BMI slope even after adjusting for maternal BMI (coefficient 0.17, representing the change in child BMI z-score over the entire study period associated with the lowest, relative to the highest, maternal education level). There was no statistically significant (p<0.05) interaction between maternal BMI and education in this model. Additional analyses found no association for family income or area deprivation with child BMI intercept or slope after adjustment for maternal BMI (see online supplementary file S1).

**Table 2:** Associations between lower maternal education and child BMI trajectory, N=2957

	Intercept (46 months)		Slope (46–94 months)	
	β (SE)	p Value	β (SE)	p Value
Stage 1—not adjusted for maternal BMI	0.07 (0.08)	0.365	0.20 (0.05)	<0.001
Stage 2—adjusted for maternal BMI	0.03 (0.08)	0.749	0.17 (0.05)	0.001

Models adjust for child gender and exact age at BMI measurements, maternal minority ethnic status. Model fit statistics: comparative fit index 0.99, root mean square error of approximation 0.05. Figures show coefficients and SEs, and represent the effect of the slope index of inequality for maternal education, which compares lowest with highest education level.

BMI, body mass index.

10.1136/jech-2015-206616.supp1 Supplementary data

**Figure 2:**
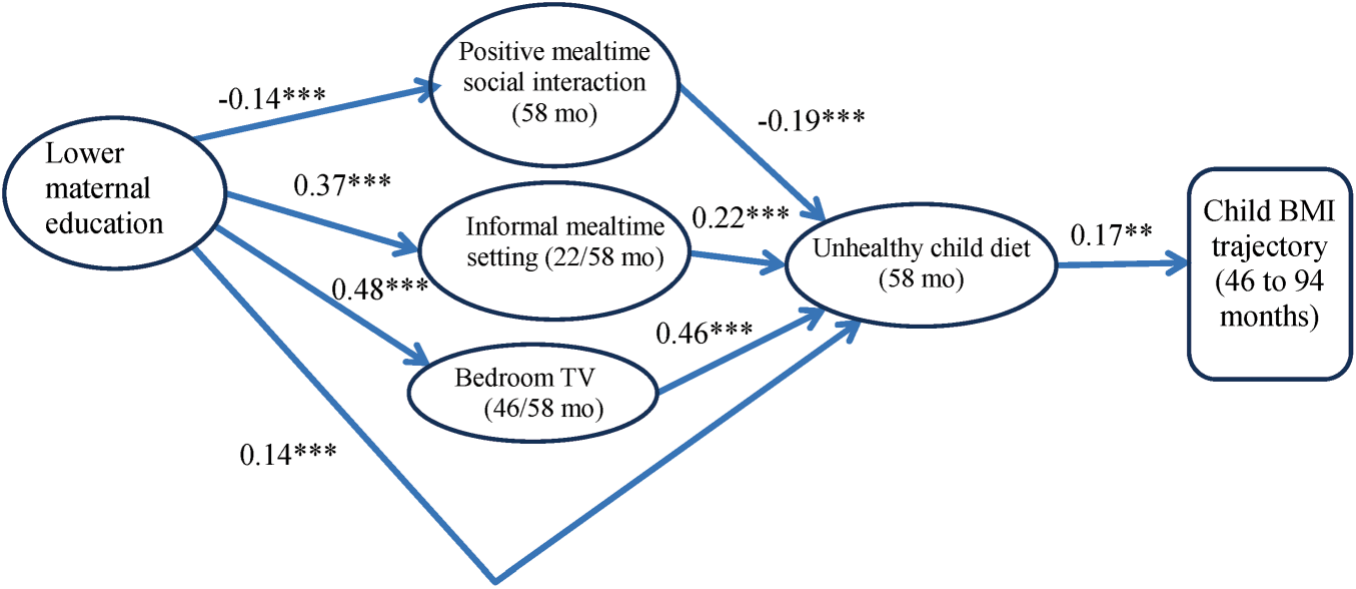
Path model of associations between maternal education and BMI slope via parenting and unhealthy diet, N=2957. Note: model shows significant pathways only, with standardised coefficients (**p<0.01, ***p<0.001). Model adjusts for child gender and exact age at first BMI measure, maternal BMI and minority ethnic status. BMI, body mass index; mo, months.

A measurement model was constructed to assess the four latent constructs (unhealthy diet, informal meal setting, positive mealtime interaction and bedroom TV) used as potential mediators of associations between maternal education and child BMI trajectory. Two indicators of unhealthy diet (soft drink and fruit consumption) with loadings <0.4 were dropped. Further details of this model are provided in an online supplementary file S2. Figure 2 shows the results of the path model used to explore associations between maternal education and child BMI slope via the four latent constructs. For simplicity, this figure is restricted to statistically significant pathways (p<0.05), and to aid comparability, standardised estimates of coefficients have been provided. All significant pathways from parenting measures to child BMI were via unhealthy diet. Lower maternal education predicted less positive mealtime interaction (−0.14), but greater likelihood of informal mealtime setting (0.37) and bedroom TV (0.48). In turn, positive mealtime interaction was negatively associated with unhealthy diet (−0.19), while informal mealtime and bedroom TV both had positive associations (0.22 and 0.46, respectively). There was also a positive effect from lower maternal education to unhealthy diet, not via parenting (0.14). Finally, unhealthy diet had a positive association with child BMI slope (0.17).

10.1136/jech-2015-206616.supp2 Supplementary data

Estimates of indirect, direct and total effects of lower maternal education on child BMI slope are shown in table 3.

**Table 3:** Direct and indirect effects of lower maternal education on child BMI slope

	Estimate (95% CI)	p Value
Total	0.18 (0.07 to 0.28)	0.001
Total indirect	0.16 (0.07 to 0.25)	0.001
Specific indirect effects		
Via unhealthy diet	0.06 (0.02 to 0.12)	0.019
Via informal setting	0.02 (−0.05 to 0.07)	0.561
Via less positive mealtime interaction	−0.02 (−0.05 to 0.00)	0.066
Via bedroom TV	−0.03 (−0.15 to 0.08)	0.559
Via informal setting and unhealthy diet	0.03 (0.01 to 0.07)	0.011
Via less positive mealtime interaction and unhealthy diet	0.01 (0.00 to 0.03)	0.032
Via bedroom TV and unhealthy diet	0.09 (0.03 to 0.16)	0.005
Direct effect	0.02 (−0.13 to 0.16)	0.821

Model as for figure 2. Unlike figure 2, this table shows unstandardised estimates. Model adjusts for child gender and exact age at first BMI measure, maternal BMI and minority ethnic status.

BMI, body mass index.

The indirect effects of lower maternal education via all mediators (0.16) comprised 89% of the total effect of maternal education on BMI slope. Pathways to BMI slope from maternal education through parenting and then unhealthy diet accounted for 68% of the statistically significant indirect pathways. The main indirect pathway involving parenting was via the effect of bedroom TV, with smaller effects of informal meal setting and less positive mealtime interaction.

A sensitivity analysis to allow for the complex survey design was conducted, using mediator factor scores to replace latent variables (see online supplementary file S3). The relative magnitude of pathways was similar to findings in table 3, although as expected with the use of directly observed mediators^23^ the total indirect effect was reduced.

10.1136/jech-2015-206616.supp3 Supplementary data

## Discussion

Our findings confirm other research suggesting the emergence and growth of inequalities in mean child BMI trajectory relating to maternal education after about 4 years of age,^2^ ^3^ and extend the mainly cross-sectional evidence base.^1^ ^3^ Pathways leading to the emergence of SEP inequalities in young children's BMI trajectory have received little attention in the literature to date. This is the first study to suggest that a substantial part of these inequalities may be understood in terms of the effect of parenting related to the physical and social context of food consumption on children's unhealthy diet.

Two indirect pathways via informal meal setting and bedroom TV suggest the likely influence of TV on children's eating behaviour. TV food advertising acts as a direct stimulus to consumption of unhealthy snacks.^29^ More generally, watching TV while eating interferes with normal habituation to food cues, delays feelings of satiety and reduces memory of food consumed.^30^ Our informal meal setting measure included eating main meals in front of a TV, and echoes other research finding a link between this behaviour and increased child BMI in North America and Europe.^12^ ^14^ ^31^ A pathway to BMI from bedroom TV via unhealthy diet was also suggested in one of these European studies:^14^ both this and longitudinal US research on 10–14-year-olds^32^ found effects of eating main meals while watching TV and bedroom TV on BMI, even after controlling for hours of TV. This appears to tally with our own finding of no significant direct path from our two TV-related mediators to child BMI, as would be expected if the effect was through more sedentary behaviour or less sleep. Since few children in our study ate their main meal in their bedroom, our finding for a path from bedroom TV to BMI via unhealthy diet may reflect greater consumption of energy-dense snacks while watching TV.^12^ ^14^ ^33^ Like others,^32^ we speculate that bedroom TV might directly stimulate snack consumption, via the child selecting TV programming incorporating greater exposure to unhealthy food advertising. Additional effects of bedroom TV on greater consumption of energy-dense foods might relate to lower parental control of bedroom activities, as research has shown weaker links between authoritative parenting and sugary drink consumption among children with a bedroom TV.^34^ Bedroom TV might also delay children's sleep onset, which has been linked to poor diet quality and BMI,^35^ although causal relationships between TV, sleep and diet remain uncertain. We were unable to explore other factors that may be confounded with bedroom TV and unhealthy diet, such as family physical activity and access to recreational facilities.

Additional pathways less directly related to TV use are suggested by our study, involving mealtime setting and social interaction. The role of informal setting, away from a dining area, is echoed by cross-sectional research linking eating in the kitchen or dining room to lower child BMI.^16^ Our effect of setting might be confounded with omitted variables such as material hardship, although BMI inequalities were associated with neither income nor area deprivation. Effects may relate to lower parental monitoring of food intake and/or less insistence on eating at particular times.^36^ We also found a small pathway via less positive social interaction, in agreement with another study associating positive mealtime communication with lower child BMI,^11^ and supporting the idea that positive parental communication at mealtimes promotes healthy eating.^36^ Nonetheless, it is not possible to rule out other aspects of parenting that may be confounded with this pathway.

### Limitations

Although suggested pathways appear plausible from what is known elsewhere, detailed elements require further investigation to substantiate causal mechanisms. Part of the effect of maternal education remained unaccounted for by our parenting mediators, and additional pathways underlying the development of inequalities in BMI trajectory (eg, via parental modelling of diet, or feeding style) require investigation. Our study has several other limitations. We only had child BMI information for three time points, restricting us to a linear latent growth curve model; and modelling maternal education as a slope index assumed a linear relationship with BMI. With the exception of height and weight measurements for child and mother, we relied on maternal reports. The Growing Up in Scotland study is a multipurpose study: thus, many measures are indicative, rather than comprehensive. Maternal BMI was not measured until the child was aged 70 months: future studies should incorporate earlier measures of maternal and child BMI over a longer period. Strengths of the study include use of a large, longitudinal sample that was nationally representative at baseline, incorporating weights to compensate for differential attrition, and use of a latent variable mediator approach.^23^

In conjunction with the widespread phenomenon of high rates of childhood obesity over recent decades, there is evidence for growing socioeconomic inequalities.^37^ ^38^ To date, there is only scanty evidence for the effectiveness of interventions designed to reduce these inequalities, although there are indications that restricting TV time could be effective.^39^ While further work on mealtime social interactions is needed, to capture more detailed information, our findings suggest aspects of parenting, especially provision of bedroom TV and informal meal setting for children, may be suitable intervention targets.

**What is already known on this subject**

- Inequalities in body mass index (BMI) relating to maternal educational level start to emerge when children are about 4 years old.
- Pathways underlying the development of these inequalities are not well understood.

**What this study adds**

- The study modelled pathways from maternal education to young children's BMI trajectory via parenting and unhealthy diet. It focused on three aspects of parenting more commonly reported by mothers with lower, rather than higher, education levels: provision of bedroom TV for the child; informal main meal setting and less positive mealtime social interaction.
- A substantial part of the inequalities in child BMI trajectory associated with maternal education could be interpreted in terms of pathways from lower maternal education via these three aspects of parenting to unhealthy child diet, and in turn to child BMI trajectory.
- Interventions targeting these three aspects of family life might reduce inequalities in young children's BMI trajectories associated with maternal education.

## Footnotes

i See http://www.scqf.org.uk/TheFramework/. At the time of the survey, higher and standard grades were the respective Scottish equivalents of advanced level and general certificate of education qualifications obtained in other parts of the UK (England, Wales and Northern Ireland).

ii No gender difference was observed.
