# Supplementary material for: Does parenting help to explain socioeconomic inequalities in children's body mass index trajectories? Longitudinal analysis using the Growing Up in Scotland study
Source: J Epidemiol Community Health. 2016 Apr 7;70(9):868–73. doi: 10.1136/jech-2015-206616 (PMC5013155; doi:10.1136/jech-2015-206616)
Supplement: Supplementary data [file jech-2015-206616supp_S1.pdf]

Supplemental File S1

Analysis of associations between additional measures of parental socio-economic position (SEP) and child BMI trajectory

Measures of SEP

Family income was based on maternal reports at child aged 46 months in response to a question about the gross (pre-tax) level of total family income from all sources, including employment income, welfare benefits and interest from savings, with categorical response options depending on whether the respondent chose to report weekly, monthly or annual amounts (for example, for annual amounts there were 17 options ranging from less than £3,999 to £56,000 or more). Annual amounts were calculated and equivalised using the McClements Scale to take into account the effects of the number of children, and the ages of the children, on the living standards of the household.

Area deprivation used residential postcode information to ascertain the Scottish Index of Multiple Deprivation (SIMD) quintile score at child aged 46 months. The SIMD identifies concentrations of multiple deprivation across Scotland by assigning a score to small census areas or “data zones” (median population around 750), derived from 37 indicators across 7 domains: current income, employment, health, education, skills and training, housing, geographic access and crime. Quintile scores rank deprivation from 1 (least deprived 20%) to 5 (most deprived 20%).

A slope index of inequality, SSI (Low and Low 2004) was calculated for each measure of family SEP. The index of inequality takes account of how a measure is distributed in the population, by using rankings of SEP. Rankings were scaled in reverse order, with zero representing the *highest*-ranking SEP and one the *lowest* possible SEP rank. The SSI coefficient represents the difference in outcome when the lowest SEP rank is compared with the highest.

Associations between additional measures of family SEP and child BMI trajectory, N=2957

		Stage 1 - not adjusted for maternal BMI				Stage 2 - adjusted for maternal BMI			
		Intercept (46 months)		Slope (46 to 94 months)		Intercept (46 months)		Slope (46 to 94 months)	
**SEP measure – slope index of inequality**	Direction of effect	β (SE)	*p*	β (SE)	*p*	β (SE)	*p*	β (SE)	*p*
Family income	lower	0.15 (0.08)	0.059	0.11 (0.05)	0.054	0.10 (0.08)	0.237	0.07 (0.06)	0.233
Area deprivation	higher	0.06 (0.07)	0.365	0.11 (0.05)	0.016	0.00 (0.07)	0.920	0.07 (0.05)	0.146

Note: Models adjust for child gender and exact age at BMI measurements, maternal minority ethnic status. SEP measures are not mutually adjusted. Figures show coefficients and standard errors. For each SEP measure, the slope index of inequality shows the effect of moving from the highest to the lowest SEP.
