# Supplementary material for: Does parenting help to explain socioeconomic inequalities in children's body mass index trajectories? Longitudinal analysis using the Growing Up in Scotland study
Source: J Epidemiol Community Health. 2016 Apr 7;70(9):868–73. doi: 10.1136/jech-2015-206616 (PMC5013155; doi:10.1136/jech-2015-206616)
Supplement: Supplementary data [file jech-2015-206616supp_S2.pdf]

Supplementary file S2

Measurement model of latent constructs of unhealthy child diet and parenting

**Unhealthy child diet**	**β (SE)**	**Loading**
Sweets/chocolate (58 months)	0.48 (0.02)	0.48
Crisps (58 months)	0.47 (0.03)	0.47
Vegetables (58 months)	-0.42 (0.02)	-0.42
Skip breakfast (58 months)	0.55 (0.06)	0.55
**Informal meal setting**		
Main meal in non-dining area (22 months)	0.75 (0.02)	0.78
Main meal in non-dining area (58 months)	0.92 (0.02)	0.92
Eat main meal watching TV (58 months)	0.63 (0.02)	0.62
**Positive mealtime social interaction**		
Mealtimes are enjoyable (58 months)	0.54 (0.03)	0.54
Mealtimes give us time to talk (58 months)	0.81 (0.04)	0.81
**Bedroom TV**		
Bedroom TV (46 months)	0.92 (0.02)	0.92
Bedroom TV (58 months)	0.99 (0.02)	0.99

Correlations among latent constructs

	**Unhealthy child diet**	**Informal meat setting**	**Positive mealtime social interaction**
**Unhealthy child diet**			
**Informal meal setting**	0.52		
**Positive mealtime social interaction**	-0.35	-0.39	
**Bedroom TV**	0.65	0.50	-0.22

Notes: Model fit Comparative Fit Index =0.99, Root Mean Square Error of Approximation =0.03. Standardised coefficients (β) and standard errors (SE) are shown, all p<0.001.
