# Supplementary material for: Does parenting help to explain socioeconomic inequalities in children's body mass index trajectories? Longitudinal analysis using the Growing Up in Scotland study
Source: J Epidemiol Community Health. 2016 Apr 7;70(9):868–73. doi: 10.1136/jech-2015-206616 (PMC5013155; doi:10.1136/jech-2015-206616)
Supplement: Supplementary data [file jech-2015-206616supp_S3.pdf]

Supplementary file S3
Sensitivity analysis - effects using mediator factor scores
Direct and indirect effects of lower maternal education on child BMI slope

	Estimate (SE)	*p*
**Total**	0.17 (0.05)	0.001
**Total indirect**	0.10 (0.03)	0.002
**Specific indirect effects**		
Via unhealthy diet	0.01 (0.01)	0.078
Via informal setting	0.02 (0.04)	0.596
Via less positive mealtime interaction	-0.03 (0.01)	0.023
Via bedroom TV	-0.03 (0.05)	0.581
Via informal setting and unhealthy diet	0.02 (0.01)	0.014
Via less positive mealtime interaction and unhealthy diet	0.01 (0.00)	0.009
Via bedroom TV and unhealthy diet	0.09 (0.04)	0.010
**Direct effect**	0.07 (0.06)	0.232

Model as for Figure 2. Unlike Figure 2, this table shows unstandardised estimates.
